# Perspectives from healthcare professionals on the nutritional adequacy of plant-based dairy alternatives: results of a mixed methods inquiry

**DOI:** 10.1186/s40795-022-00542-7

**Published:** 2022-05-12

**Authors:** Bridget E. Clark, Lizzy Pope, Emily H. Belarmino

**Affiliations:** Department of Nutrition and Food Sciences, University of Vermont, 256 Carrigan Wing, 109 Carrigan Drive, Burlington, VT 05405-0086 USA

**Keywords:** Dairy alternatives, Dairy, Health professionals, Plant-based diets, Plant-based labeling

## Abstract

**Background:**

Healthcare professionals are important sources of nutrition and health information for Americans. As plant-based (PB) dairy alternative products increase in popularity, concerns have been raised about their nutritional adequacy, and whether consumers understand nutritional differences to dairy. Healthcare professionals directly advise consumers on dietary choices, therefore we sought to examine their understanding and opinions of PB dairy alternatives.

**Methods:**

We analyzed comments submitted to the U.S. Food and Drug Administration (FDA) by health professionals (*n* = 191) in 2018–2019 in response to a request for public comment on the nutrition of PB dairy alternatives and the use of dairy terms like “milk”, “cheese”, and “yogurt” on their labels. Survey data from healthcare professionals (*n* = 417) was collected in 2020–2021. Comments and survey responses to open-ended questions were coded using template analysis and thematically analyzed. Logistic regression models examined perceptions across health professional characteristics for close-ended survey responses.

**Results:**

Three-fourths of health professionals believe consumers are confused about the nutritional differences between dairy and PB dairy alternatives. Over half (53%) do not believe either product is nutritionally superior to the other. Many believe dairy products have higher nutrient value, but also believe PB dairy alternatives can be part of a healthful diet. Compared to other types of health professionals, dietetics professionals demonstrated a more accurate understanding of the nutritional value of both products and were more likely to believe nutrients like protein (OR 2.02; 95% CI 1.22–3.34, *p* = 0.006) and vitamin D (OR 2.46; 95% CI 1.48–4.09, *p* = 0.001) may be nutrients of concern for PB dairy alternative consumers. They were also more likely to believe consumers are confused about these products (OR 3.44; 95% CI 1.65–7.21; *p* = 0.001). Health professionals who submitted comments to the FDA showed stronger opinions in favor of PB dairy alternatives.

**Conclusions:**

Although PB dairy alternatives have nutritional value in certain diets, responses from health professionals suggest that changing their labeling to be different than dairy may reduce confusion. Improved nutrition education among health professionals may also be necessary.

**Supplementary Information:**

The online version contains supplementary material available at 10.1186/s40795-022-00542-7.

## Background

Healthcare professionals including registered dietitian nutritionists (RDN), primary care providers, dentists, dental hygienists, and nurses provide nutrition care and advice to Americans, and many view these professionals as trustworthy and credible sources of nutrition and health information [1, 2, 3]. Research shows that healthcare providers’ nutrition guidance can positively impact patient dietary behavior [4]. One dietary trend that health providers may be asked about is the replacement of animal-source foods with plant-based (PB) alternatives. Although the number of Americans identifying as vegans and vegetarians is small, about half of Americans show willingness to consume a diet lower in animal-source foods [5]. Over the past decade, demand for PB dairy alternatives, including non-dairy “milk,” “cheese,” and “yogurt,” has increased significantly [6, 7]. At the same time, dairy milk consumption per person in the U.S. has decreased by almost 50% since 1975 [8], and the percentage of households purchasing dairy milk at all continues to decline [9]. Consumers report choosing PB alternatives over dairy for a variety of reasons, including allergy or intolerance [10, 11], environmental concerns [5, 12], animal welfare concerns [5], and health-related factors [5, 11, 12].

The U.S. Dietary Guidelines still recommend daily dairy consumption, although over 80% of the U.S. population is not meeting the recommended intake of three servings daily [13]. Dairy milk is a top source of carbohydrates, fats, protein, and naturally contains or is fortified with important micronutrients like calcium, vitamins A and D, and potassium [12, 14]. However, dairy has received media scrutiny surrounding potential adverse health effects [15, 16, 17]. Although the value of PB beverages has been debated [17, 18, 19], these products do have desirable attributes. Most non-dairy beverages contain little or no saturated fat [14], and some are high in polyunsaturated fat [12, 20], which may improve cardiovascular disease risk [21, 22]. Non-dairy beverages also tend to be lower in calories, supporting weight maintenance, and higher in vitamin E, supporting protection against cancer-promoting free radicals [20]. Still, concerns remain about the nutritional adequacy of diets that replace dairy foods with PB alternatives [20, 23, 24]. Other than soymilk, the most popular types of PB beverages in the U.S. contain little protein [12, 14, 25, 26], and fortified soymilk remains the only PB beverage identified as a nutritionally adequate substitute for dairy in the Dietary Guidelines for Americans [13]. PB beverages are often fortified with the same micronutrients as those found in dairy, although it is unclear if these fortified micronutrients have the same bioavailability [14, 20]. Bioavailability may be reduced by certain plant compounds known as “anti-nutrients”, such as phytic acid, found in some PB products that can bind to minerals and disrupt their absorption [20]. It is important for consumers of PB dairy alternatives to replace missing nutrients in other areas of their diet [12], but many may not be aware of this.

A limited body of research has examined consumer perceptions towards PB dietary patterns and PB dairy alternatives [27, 28, 29, 30, 31] revealing that many view PB diets positively, but misconceptions exist about the nutritional properties of dairy milk and PB alternatives. As demand for PB dairy alternatives increases, ensuring accurate consumer understanding of the nutritional differences is important [12, 32, 33]. Members of the dairy industry and some nutrition experts have raised concern that labeling PB products with dairy food names like milk, cheese, and yogurt is leading consumers to believe they contain the same nutrient content as dairy [20, 34, 35, 36]. They argue PB products should not be allowed to be labeled in this way, as they do not meet the U.S. Food and Drug Administration’s (FDA) standards of identity for milk products [37]. In January 2017 the National Milk Producers Federation (NMPF) helped introduce The DAIRY PRIDE Act to Congress, which called to prohibit PB products from using dairy terms in their packaging [34]. In part prompted by this, in September 2018, the FDA requested public comment on issues related to the current labeling of PB substitute products, to gather information on consumer understanding about the differences between dairy and PB dairy alternatives [38].

As PB dietary patterns grow, it is important to understand what health professionals – particularly those who directly counsel patients – understand and believe about PB products. Yet, to date, there is limited documentation of this. In a study investigating beliefs influencing dairy versus PB dairy alternative consumption using focus groups with 161 Canadian adults, healthcare provider approval or disapproval was stated as a major influence of milk and cheese product consumption by several participants [39]. Research conducted in the U.S. has found a high level of support from RDNs for PB protein consumption [40], and most RDNs and dietetics students support PB dietary patterns [41, 42]. A survey of medical doctors found that the majority believed PB diets were health-promoting, while only one-third reported a willingness to recommend a PB diet to their patients [43]. In another study of nutrition beliefs among health professionals, nearly two-thirds agreed that PB diets were beneficial to health, and over half felt that overconsuming dairy was harmful to health, although many also agreed that dairy was an important source of calcium [44]. A small study including 15 physicians found mixed beliefs on dairy fat recommendations for children [45]. However, no studies to date have examined health professionals’ perceptions of dairy compared to PB alternatives. Likewise, no studies have examined Federal Register comments submitted specifically by healthcare professionals on a health debate.

Given that they are key sources of nutrition information for the U.S. consumer, and their advice may directly influence consumer food choice, health professionals’ views regarding PB alternatives are important to assess. In this cross-sectional study, we examined health professionals’ knowledge and perceptions of PB dairy alternatives and their labeling in the U.S., and examined knowledge differences between dietetics professionals and all other health professionals.

## Methods

### Sample

The study sample consisted of (1) health professionals who participated in the FDA’s request for comment on the labeling of PB products with dairy food names such as “milk,” “cultured milk,” “yogurt,” and “cheese” [38] (83 FR 49,103 2018), and (2) a national sample of health professionals who completed an online survey on this topic.

### Federal Register comments

The Federal Register is a U.S. government daily publication of Federal agency rules, proposed rules, and public notices. Documents posted in the Federal Register are open to public comment [46]. In response to the FDA’s request for comment on PB labeling posted on September 28^th^, 2018, a total of 11,906 comments were submitted to the Federal Register before the deadline on January 28^th^, 2019. All comments submitted by the deadline were obtained from the FDA through a Freedom of Information Act request. Comments and commenters’ self-identified characteristics were imported into the NVivo qualitative data analysis software (version 12). Duplicate submissions (including petitions) with no unique text were identified and only one of each duplicate was retained for analysis. Off-topic and unusable submissions, as well as submissions from minors, were removed from the dataset. Following the removal process, 8,052 comments remained for analysis (Additional File 1).

As part of the standard process, submitters were asked to select their country and state or province of residence, as well as their submission category. Submission category options included individual consumer, food industry, health professional, and private industry, among others. We combined the categories to create six groupings: individual consumers (including consumer group representatives and international public citizens), representatives of a food industry or association (food industry, food association, international food association), government representatives (federal, state, local, international, and other), health professionals, members of academia, and representatives of another industry or association (including any other U.S. or international industry or association listed). Within their comment, some submitters also self-identified as health professionals, by listing their degree qualifications or indicating that they are a health professional. We identified 191 commenters as a health professional from the body of their comment (*n* = 71) and/or if they chose the “health professional” category during submission (*n* = 154) (Additional File 1).

### Survey

We designed a web-based (Qualtrics, Provo, UT) survey to measure health professionals’ knowledge and opinions of dairy products and PB alternatives. To be eligible, individuals had to be age 18 years or older; live in the U.S. since at least January 2020; and currently be an RDN, medical doctor (MD), doctor of osteopathy (DO), physician assistant (PA), dentist, dental hygienist, licensed practical nurse (LPN), registered nurse (RN), nurse practitioner (NP), or a student currently enrolled in a degree program for one of those professions holding Junior undergraduate standing or above. These specific categories of health professionals were targeted as likely to have the most opportunity to be asked for and to provide nutrition advice to patients. A screener was included at the start of the survey which confirmed these eligibility guidelines were met before the respondent could continue with the survey. We used three methods for non-probability convenience sampling to recruit respondents: (1) paid digital ads via Facebook to reach a national sample; (2) targeted postings to relevant national LinkedIn and Facebook group pages for health professionals; and (3) postings on academic, professional, and community listservs in Vermont as well as nationally. The survey was open from November 19, 2020 until February 4, 2021. All respondents who passed the eligibility screener and completed all or part of the survey were included in analyses. A total of 417 survey responses were retained for analysis. Respondents were entered in a gift card drawing. Both portions of this study were determined to be exempt from review by the Committee on Human Research in the Behavioral and Social Sciences of the Institutional Review Board at the University of Vermont.

The survey asked respondents’ age, gender identity, race, ethnicity, state of residence, and type of health profession or professional degree program. The survey also measured respondents’ perceptions of the nutritional content of dairy versus PB alternatives, and of the labeling of dairy and PB alternatives (Table 1). These questions were developed based on those asked by the FDA in their request for comments [38]. Specifically, it asked which products, dairy or PB alternatives, if either, they believe are nutritionally superior over the other (dairy products, plant-based products, or neither), and why, and which nutrients may be below adequate intake in individuals who consume PB alternatives in place of dairy (calcium, potassium, protein, vitamin A, vitamin D or none), and why. It also asked respondents’ opinions on consumers’ reasons for choosing PB alternatives (nutrition and health concerns, animal welfare concerns, environmental concerns, dairy allergy or intolerance, food safety concerns, hormone and/or antibiotic concerns, taste preferences, or other), if consumers understand the nutrition of PB dairy alternatives (yes, no, or unsure), if the labeling of these products affects consumer understanding (yes, no, or unsure), and whether the FDA should permit PB products to be labeled with names that include the names of dairy foods (yes, no, or unsure). Additionally, the questionnaire asked respondents if they had ever submitted a comment to the Federal Register, and if so, if they had submitted a comment to this request from the FDA. Lastly, it asked respondents if there was anything else they would like to share about the labeling of PB dairy alternatives.

**Table 1:** PB dairy alternative survey questions

Variable name	Question
Submitted to Federal Register	Have you ever submitted a comment to the U.S. Federal Register?
Submitted to FDA request	Did you participate in in the Food and Drug Administration’s request for comments regarding the labeling of plant-based products with names that include the names of dairy foods such as “milk,” “cultured milk,” “yogurt,” and “cheese”? ^a^
Consumer reasons	What do you believe are consumers’ reasons for purchasing and consuming plant-based milk products (milk products not from animal origin) that are labeled with the names of dairy foods such as “milk,” “cultured milk,” “yogurt,” and “cheese”? Please select all that apply.
Nutritionally superior product	Which product (plant-based milk or dairy milk products), if either, do you believe is nutritionally superior to the other?
Why nutritionally superior product	Please explain the reasons why you believe [dairy OR plant-based OR neither] products are nutritionally superior. ^b^
Nutrients of concern	Do you believe that the diets of people who consume mostly plant-based milk products are lacking in any of the following nutrients compared to the diets of people who consume mostly dairy products? Please select all that apply.
Why nutrients of concern	Please explain why you selected your answer(s) above.
Consumer understanding	Do you believe consumers understand the nutritional differences between plant-based products and their dairy counterparts?
PB label effect	Do you think the use of dairy names such as “milk,” “cultured milk,” “yogurt,” and “cheese” on plant-based product labels affects consumers’ perceptions and understanding of these products?
Allow dairy terms on PB labels	Do you think that the Food and Drug Administration should permit plant-based products to be labeled with names that include the names of dairy foods such as “milk,” “cultured milk,” “yogurt,” and “cheese”?
Other milk comments	Is there anything else that you would like to share about the labeling of plant-based milk products?

^a^ Only asked if respondent selected yes to previous question. ^b^ Not asked to respondents who selected ‘I am unsure’ to nutritionally superior product question

### Coding Federal Register comments and survey responses

We used the template analysis approach [47, 48] to develop a codebook including a priori codes and codes that reflected emergent themes. The coding team iteratively applied the template to subsets of the public comment data in June 2019 and met to resolve differences in interpretation and revise the template. The final template consisted of two categories of codes: characteristic codes (relevant to the Federal Register comments only) and content codes. Characteristic codes captured self-described characteristics of commenters, including their affiliation (e.g., representative of an organization), their preference for dairy products or PB alternatives, and their position on the labeling of PB products with dairy terms. Content codes captured main themes in the data, including discussion of nutrition and health topics. See Additional File 2 for codes relevant to this work. Coding of the full public comment dataset was divided amongst an eight-person team. Each coder’s ability to consistently the codebook was checked by the corresponding author for a sample of data prior to participation in coding. Open-ended survey responses were coded using the same codebook and methods. After coding was complete, our research team checked each code for accuracy and consistency in use. Coding anomalies were discussed and corrected.

### Data Analysis

#### Federal Register analysis

Federal Register comments from health professionals were analyzed using thematic analysis, a method used to identify, organize, and describe key findings from a qualitative data set [49]. Frequencies of all relevant codes (Additional File 2) were tabulated and reviewed. Key themes were summarized in code memos, which were reviewed and refined by the research group. For qualitative analysis of both samples, the frequency of codes and themes discussed by participants is reported using the following: “almost all” indicates ≥ 90%; “most” means more than two-thirds; “many” indicates between half and two-thirds; “about half” means about 50%; a “substantial minority” indicates at least one-third yet less than half; “some” means less than a third; and “few” or “a small number” means ≤ 10% of submissions [50]. A chi-square test was used to examine differences in positions on the labeling of PB products with dairy terms between health professional and non-health professional commenters. The test was statistically significant if *p* < 0.05.

#### Survey analysis

Open-ended survey responses were thematically analyzed using the same methods described above for analysis of Federal Register comments. Matrix coding queries were used to consider responses to open-ended questions between dietetics professionals (including dietetics students) and all other health professionals (including other health professional students). Quantitative survey data was analyzed with IBM SPSS Statistics (version 27). During analysis, variables for race and ethnicity were merged and recoded to a binary variable “Black, Indigenous, or person of color (BIPOC)” and “not BIPOC”. State of residence was recoded to a binary variable that indicated whether it was a “dairy state”. U.S. “dairy states” were determined based on being among the 10 states with the highest percentage of total farm sales coming from milk sales in 2017 (VT, NM, NY, WI, ID, NH, PA, AZ, MI, and ME) [51]. Also, the nutritionally superior product variable was recoded to combine the responses “I believe neither is nutritionally superior to the other” and “I am unsure”. Univariate descriptive statistics were generated for all relevant variables. Chi-square tests were used to examine bivariate associations of dairy and PB dairy alternative perceptions between dietetics professionals and other health professionals. We used unadjusted and adjusted logistic regression models to examine whether health profession (dietetics professionals versus other health professionals) was associated with each of the following outcomes: respondents’ beliefs on which product was nutritionally superior, nutrients of concern, consumer understanding, PB label effect, and allowing dairy terms in PB labeling. Demographic factors including age, race/ethnicity, and whether or not the respondent lived in a dairy state were included in adjusted models. Tests were statistically significant if *p* < 0.05.

## Results

Of the 8,052 original comments received by the FDA in response to this request for comment, less than 2.5% (*n* = 191) were submitted by health professionals (Table 2). A little more than half of health professional commenters (55.5%) reported living in the U.S., with the majority also specifying their state of residence. Nearly one in ten (9.4%) of these health professional commenters reported residing in a “dairy state”. The remainder reported a location outside of the U.S. (0.5%) or did not report a location (44.0%).

**Table 2:** Characteristics of Federal Register commenters

Variable	Total commenters		Health professionals		Non-health professionals	
	*n*	%	*n*	%	*n*	%
Location						
United States	3779	46.9	106	55.5	3673	46.7
Dairy State	776	20.5	10	9.4	766	20.9
Other State	2623	69.4	83	78.3	2540	69.1
Did not specify	380	10.1	13	12.3	367	10
Other country	150	1.9	1	0.5	149	1.9
Did not specify	4123	51.2	84	44.0	4039	51.4
Product preference						
Prefer dairy products	599	7.4	21	11.0	578	7.3
Prefer PB products	4000	49.7	81	42.4	3919	49.9
Unclear or neutral preference	3453	42.9	89	46.6	3364	42.8
Position on labeling						
Support use of dairy terms in PB labeling	5674	70.4	124	64.9	5550	70.6
Oppose use of dairy terms in PB labeling	1021	12.7	47	24.6	974	12.4
Unclear or neutral position on PB labeling	1357	16.9	20	10.5	1337	17.0

*N* = 8052, *n* = 191 for health professionals, *n* = 7861 for non-health professionals

Half of non-health professional commenters (49.9%) and 42.4% of health professional commenters described a preference for PB dairy alternatives. Only 7.3% of non-health professional commenters and 11.0% of health professionals indicated a preference for dairy products. A substantial portion of non-health professional commenters (42.8%) and health professional commenters (46.6%) did not indicate a preference for dairy vs. dairy alternatives. Compared to non-health professional commenters, a smaller proportion of health professionals supported the use of dairy terms in PB product labels (68.9% vs. 64.9%; X^2^ = 27.53, *p* < 0.001; data not shown).

Most survey respondents were female (91.5%), non-Hispanic white (87.5%), and under age 55 (82.2%) (Table 3). Forty-six percent were from a “dairy state”, reflecting the additional recruitment conducted in Vermont. The sample consisted of 350 practicing health professionals and 67 students hereafter collectively referred to as “health professionals”. RDNs and dietetics students made up 44.4% of the sample, and other health professionals or students made up the remainder. Nurses or nursing students comprised the largest population of other health professionals (74.1%; data not shown).

**Table 3:** Characteristics of survey respondents

Variable	*n*	%
Age		
18–34	181	43.4
35–54	161	38.6
Over 55	75	18.0
Female	301	91.8
Non-Hispanic white ^a^	275	87.9
Location		
Dairy state	150	46.0
Other state	176	54.0
Health profession		
Dietetics professional or student	185	44.4
Other health professional or student	232	56.6

*N* = 417. Totals for gender identity (*n* = 328), race/ethnicity (*n* = 313), and location (*n* = 326) are smaller because response to these questions was optional

^a^ The small number of BIPOC respondents inhibited the disaggregation of analyses by race/ethnicity

### Key nutrition and health themes of dairy and PB alternatives discussed in Federal Register comments and open-ended survey responses

*Federal Register comments*. Sixty-four percent (*n* = 123) of health professionals who commented on the Federal Register mentioned nutrition and health aspects of dairy and/or PB dairy alternatives within their comment, either describing the nutritional merits or demerits of PB dairy alternatives. Interestingly, of health professionals who supported the use of dairy terms on PB labels, 57% commented on their nutrition and health aspects, compared to 87% of those who opposed the use of dairy terms on PB labels. In order, the most common nutrition and health topics mentioned by health professionals were the general nutrition or healthfulness of either product; micronutrient content, including mentions of calcium, vitamin D, and potassium; protein content; and other nutrition or health aspects, such as chronic disease, allergy and digestion. Additionally, a substantial minority of health professionals mentioned that they believe consumers are confused about the nutritional differences between products, and some mentioned that they believe current labeling is contributing to this confusion. Illustrative quotes for each theme and subtheme are presented in Table 4.

**Table 4:** Key themes discussed in comments submitted to the FDA

Theme	Subtheme	Example quotes
Nutritional merits of PB dairy alternatives	1.PB dairy alternatives are a healthy choice	*“These are healthy alternatives with superior nutritional quality.”*
		*“The general public has finally been educated about plant-based milk being healthier for humans…”*
	2.PB dairy alternatives provide an adequate source of essential micronutrients for individualized dietary needs	*“Its still a great calcium source, and its useful for those (especially kids) who cant tolerate or dont like cows milk.”*
		*“Take any one of my patients with high cholesterol; I will tell them to choose some dairy alternatives, fortified with those essential nutrients, including vitamins and minerals…”*
	3.Dairy is harmful to human health	*“Animal products are directly linked with hypercholesterolemia, a major contributor to cardio-vascular disease, morbidity and mortality.”*
		*“Healthcare professionals like myself are making strong inroads into convincing the public of the deleterious health effects of the ingestion of any form of animal products…”*
Nutritional demerits of PB dairy alternatives	1.Dairy and PB alternatives do not have equal nutritional profiles	*“Every week, patients tell me they are using almond milk or some other nut milk as an alternative to dairy, with no clue that these are not nutritionally equivalent products.”*
		*“There is a substantial difference in the nutritional content of these two products, and consumers are understandably confused by the name "milk" on non-dairy products.”*
	2.Dairy provides a superior source of essential micronutrients	*“We know from extensive research on the benefits of dairy milk including calcium, potassium, and vitamin D, in addition to many other important micronutrients.”*
		*“I have cared for too many patients that have fractures that could have been prevented by a higher calcium intake from dairy products. The plant based beverages do not contain naturally occurring calcium…”*
	3.The protein content in dairy is superior to PB alternatives	*“As a dietitian it saddens me to see parents giving rice or coconut milk to children… less than 1 gm protein.”*
		*“The nutritional value of nut milk isn’t the same as cows milk, and the biggest difference is in the protein content. Most nut milks are not a good source of protein, and people think they are getting the same amount of protein that they would in cows milk.”*

Among those who mentioned nutrition and health, 62% of health professionals discussed the nutritional merits of PB dairy alternatives, and 35% of health professionals discussed the nutritional demerits of PB dairy alternatives (Table 4). A small remainder were neutral. Those who discussed the nutritional merits believed these products can be part of a healthy diet. About half of those who discussed their nutritional merits argued that PB dairy alternatives are nutritionally adequate replacements for dairy for certain individuals, such as those with an allergy, intolerance, or elevated risk for cardiovascular disease, and about half stated they are nutritionally superior to dairy. Some of these health professionals also believed that dairy is harmful to human health, contributing to chronic disease risk. Health professionals who discussed the nutritional demerits of PB dairy alternatives believed that they are not nutritionally equal and/or that dairy products are superior nutrient sources, especially in relation to protein. Few mentioned bioavailability differences.

*Survey responses*. The most common nutrition and health topic mentioned by survey respondents was nutrient value, specifically protein and calcium. Other top nutrition and health aspects discussed include the general nutrition or healthfulness of either product and other nutrition and health issues such as chronic disease and inflammation. Table 5 presents representative quotes for each theme. Many comments discussed the high nutrient value of dairy, most mentioning that dairy has a higher nutrient content and/or quality when compared to PB alternatives (Table 5). Almost all comments on protein described dairy as a good source of protein, with many stating dairy’s protein quality is superior to PB alternatives. Many comments that mentioned calcium stated that dairy provides more calcium than PB alternatives, some stating that calcium from PB sources is less bioavailable. Less than one-third of comments discussed the nutritional merits of PB alternatives in terms of protein, calcium, and overall nutrient content, often referencing the fortification of PB products. Few comments stated that dairy consumption is associated with disease risk.

**Table 5:** Key themes discussed among survey respondents

Theme	Example quotes
Dairy has a superior overall nutrient value compared to PB dairy alternatives	*“cows milk has a standard of identity and assures the same nutrient content regardless of brand, city, state it was produced in; plant based beverages nutrient content varies by brand and only certain brands of soy beverages come close in matching the nutrient content of cows milk”*
	*“I think that even fortified plant-based products don't contain the same amount of calcium as dairy products and because dairy products are also naturally high in 8 other essential nutrients.”*
The protein content and quality in dairy products is superior to PB dairy alternatives	*“Most plant-based beverages are very low in protein as well (for example, almond milk) which is important for children and elderly as it is common for these age groups to struggle with protein intake. Cow’s milk has more of the building block proteins that humans need in their diet.”*
	*“Dairy products tend to be a higher source of quality protein…”*
Both products have nutritional advantages and disadvantages	*“They both have nutritional benefits and deficits. I think the determining factor is the specific patients dietary needs/restrictions”*
	*“I think each have different nutrition facts. Broadly grouping them makes it hard to say they are superior. They all provide nutrients.”*
Nutritional adequacy is dependent upon the whole diet	*“Cows milk is not the sole source of vitamins and minerals. Eating a balanced diet including fruits, vegetables and whole grains will satisfy your dietary needs.”*
	*“With a healthy diet a person can get most of the nutrients needed. Plant based or not.”*

Themes were not separated between those discussing the nutritional merits vs. demerits of PB alternatives due to the small number of total comments discussing nutritional merits of PB alternatives

Regarding general nutrition and healthfulness, many health professionals commented that neither product can be deemed nutritionally superior to the other, saying both have advantages and disadvantages. Of note, some respondents shared that achieving nutrient adequacy is dependent on more than just dairy product consumption, discussing that nutrients can be obtained from many other sources with a balanced diet. No major differences were identified in themes discussed by dietetics professionals compared to other health professionals.

### Survey respondents’ perceptions and concerns about nutrition quality of dairy and PB alternatives

Top reasons identified by health professionals for why some consumers choose PB dairy alternatives over dairy were nutrition and health concerns (85.0%), dairy allergy or intolerance (82.4%), and animal welfare concerns (64.9%) (Table 6). About one-third (32.8%) believed dairy products were nutritionally superior to PB dairy alternatives, and 14.2% believed PB dairy alternatives were nutritionally superior to dairy products. About half (53.0%) did not believe that either was nutritionally superior. Most (77.2%) believed that consumers do not understand the nutritional differences between dairy products and PB alternatives, and many (65.4%) also believed the use of dairy names on PB product labels affects consumers’ understanding of these products. Just one survey respondent reported submitting a comment to the FDA’s request on this topic. Responses were split on if the FDA should permit PB dairy alternatives to be labeled with the names of dairy foods, with 39.7% saying yes, 36.4% saying no, and 23.9% remaining unsure.

**Table 6:** Survey responses to PB dairy alternative questions

Variable	*n*	%
Consumer reasons ^a^		
Nutrition and health concerns	324	85.0
Animal welfare concerns	244	64.9
Environmental concerns	194	50.9
Dairy allergy or intolerance	314	82.4
Food safety concerns	32	8.4
Hormone and/or antibiotic concerns	205	53.8
Taste	131	34.4
Other	25	6.6
Nutritionally superior products		
Dairy products	122	32.8
PB products	53	14.2
Neither or unsure	197	53.0
Consumers understand nutritional differences		
Yes	23	6.8
No	260	77.2
Unsure	54	16.0
PB labels affect consumer understanding		
Yes	225	65.4
No	70	20.3
Unsure	49	14.2
Allow dairy terms on PB labels		
Yes	136	39.7
No	125	36.4
Unsure	82	23.9

Consumer reason *n* = 381; Nutritionally superior product *n* = 372; Consumer understanding *n *= 337; PB label effect *n* = 344; Allow dairy terms in PB labeling *n* = 343

^a^ Reflects frequency of respondents who selected this reason. Selecting more than one reason was possible

**Fig. 1:**
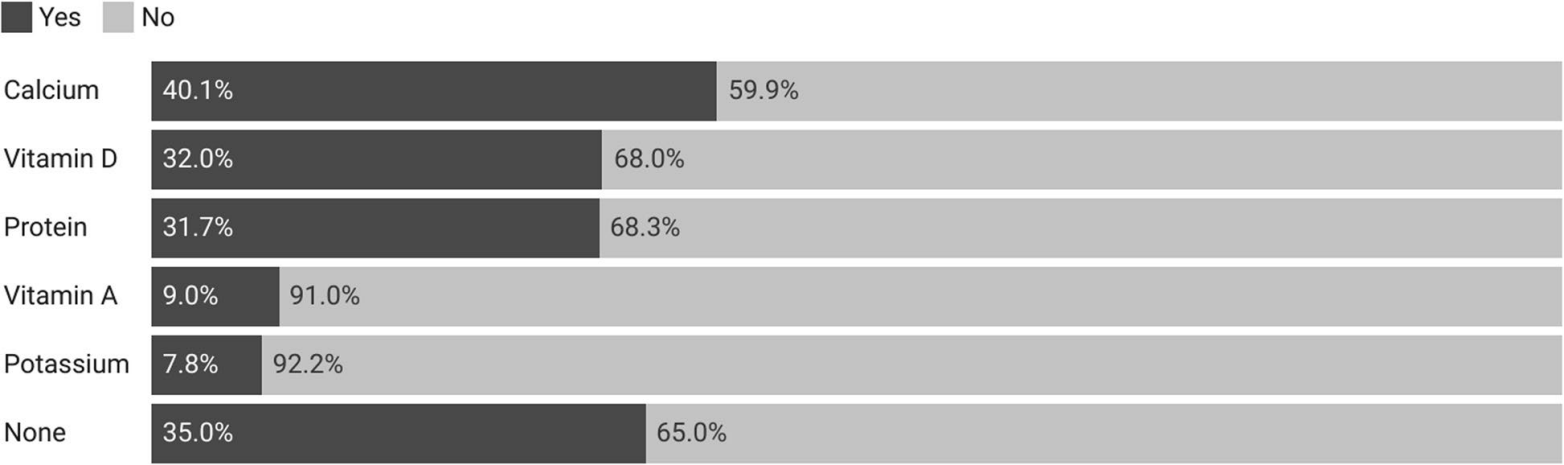
Which nutrients do health professionals believe may be of concern for PB dairy alternative consumers? (*n* = 334)

In response to the question about nutrients of concern in diets that replace dairy with PB alternatives, 40.1% of health professionals believed calcium intake may be a concern, and about one-third believed vitamin D and protein intake may be of concern (Fig. 1). Conversely, 35% of health professionals believed none of the nutrients listed were of concern in diets replacing dairy with PB alternatives.

### Perceptions of dairy and PB alternatives by health professional category

Logistic regression analyses examined if health professional type is associated with the belief that dairy or PB dairy alternatives are nutritionally superior (Table 7). Dietetics professionals were more likely than other health professionals to believe dairy is nutritionally superior to PB dairy alternatives in unadjusted (OR 1.70; 95% CI 1.08–2.69; *p* = 0.022) and adjusted models (OR 2.27; 95% CI 1.33–3.87, *p* = 0.003), and less likely to believe PB dairy alternatives are nutritionally superior to dairy in unadjusted (OR 0.26; 95% CI 0.12–0.56; *p* = 0.001) and adjusted models (OR 0.19; 95% CI 0.08–0.50, *p* = 0.001).

**Table 7:** Health professional type and belief that dairy or PB dairy alternatives are nutritionally superior (*n* = 302)

	Unadjusted analysis					Adjusted analysis				
Variable	b	SE	OR	CI	*p* value	b	SE	OR	CI	*p* value
Dairy products are nutritionally superior to PB milk products										
Dietetics professional (ref = non-dietetics professional)	0.53	0.23	1.70	1.08–2.69	**0.022**	0.82	0.27	2.27	1.33–3.87	**0.003**
PB milk products are nutritionally superior to dairy products										
Dietetics professional (ref = non-dietetics professional)	-1.35	0.39	0.26	0.12–0.56	**0.001**	-1.64	0.49	0.19	0.08–0.50	**0.001**

Unadjusted and adjusted logistic regression models are presented above. The models used “neither or unsure” as the reference category of the dependent variable. Adjusted models include age, race/ethnicity, and location (dairy state versus non-dairy state)

Logistic regression analyses also were used to identify if health professional type is associated with the belief that the diets of consumers of PB alternatives are lacking in specific nutrients (Table 8). In both unadjusted and adjusted models, dietetics professionals were more likely than other health professionals to believe protein (unadjusted: OR 1.69; 95% CI 1.06–2.69, *p* = 0.026; adjusted: OR 2.02; 95% CI 1.22–3.34, *p* = 0.006), vitamin D (unadjusted: OR 2.06; 95% CI 1.29–3.28, *p* = 0.002; adjusted: OR 2.46; 95% CI 1.48–4.09, *p* = 0.001), and potassium (unadjusted: OR 7.43; 95% CI 2.50–22.08, *p* < 0.001; adjusted: OR 7.18; 95% CI 2.35–21.95, *p* = 0.001) would be a nutrient of concern for individuals that replace dairy with PB alternatives. Conversely, they were less likely than other health professionals to believe that none of the nutrients were of concern for these individuals (unadjusted: OR 0.54; 95% CI 0.34–0.85, *p* = 0.008; adjusted: OR 0.49; 95% CI 0.29–0.81, *p *= 0.005).

**Table 8:** Health professional type and belief that diets of PB dairy alternative consumers lack specific nutrients (*n* = 299)

	Unadjusted analysis					Adjusted analysis				
Nutrient of concern	b	SE	OR	CI	*p* value	b	SE	OR	CI	*p* value
Calcium										
Dietetics professional (ref = non-dietetics professional)	0.33	0.22	1.39	0.90–2.16	0.139	0.41	0.24	1.51	0.95–2.43	0.085
Potassium										
Dietetics professional (ref = non-dietetics professional)	2.01	0.56	7.43	2.50–22.08	** < 0.001**	1.97	0.57	7.18	2.35–21.95	**0.001**
Protein										
Dietetics professional (ref = non-dietetics professional)	0.53	0.24	1.69	1.06–2.69	**0.026**	0.70	0.26	2.02	1.22–3.34	**0.001**
Vitamin A										
Dietetics professional (ref = non-dietetics professional)	0.63	0.39	1.88	0.87–4.03	0.106	0.75	0.41	2.13	0.96–4.76	0.067
Vitamin D										
Dietetics professional (ref = non-dietetics professional)	0.72	0.24	2.06	1.29–3.28	**0.002**	0.90	0.26	2.46	1.48–4.09	**0.001**
None										
Dietetics professional (ref = non-dietetics professional)	-0.63	0.24	0.54	0.34–0.85	**0.008**	-0.72	0.26	0.49	0.29–0.81	**0.005**

Unadjusted and adjusted logistic regression models are presented above. The models compared respondents who did not select each nutrient/response option to respondents who did, using not selecting that nutrient/response as the reference category of the dependent variable. Adjusted models have been adjusted for age, race/ethnicity, and location (dairy state versus non-dairy state)

Logistic regression analyses also identified if health professional type is associated with the belief that consumers understand the nutritional differences between dairy products and their PB counterparts (Table 9). Compared to other health professionals, dietetics professionals were more likely to say that consumers do not understand the nutritional differences between dairy products and their PB counterparts in both unadjusted (OR 2.72; 95% CI 1.43–5.18, *p* = 0.002) and adjusted (OR 3.44; 95% CI 1.65–7.21; *p* = 0.001) models. Results of analyses examining factors associated with beliefs on whether the use of dairy terms in PB labeling affects consumer understanding and whether the FDA should permit PB products to use dairy names in their labeling can be found in Additional File 3.

**Table 9:** Health professional type and belief that consumers understand the nutritional differences between products (*n* = 299)

	Unadjusted analysis					Adjusted analysis				
Consumer understanding	b	SE	OR	CI	*p* value	b	SE	OR	CI	*p* value
Yes										
Dietetics professional (ref = non-dietetics professional)	0.13	0.55	1.14	0.39–3.31	0.813	0.25	0.58	1.29	0.41–4.03	0.663
No										
Dietetics professional (ref = non-dietetics professional)	1.00	0.33	2.72	1.43–5.18	**0.002**	1.24	0.38	3.44	1.65–7.21	**0.001**

Unadjusted and adjusted logistic regression models are presented above. The models used “unsure” as the reference category of the dependent variable. Adjusted models have been adjusted for age, race/ethnicity, and location (dairy state versus non-dairy state)

## Discussion

This paper was the first to examine healthcare professionals’ perceptions on the nutritional value and labeling of PB dairy alternatives, and the first to use the U.S. Federal Register to examine health professionals’ opinions on a national issue. Sales of PB products are rising in the U.S. [6, 7, 52], and national and international dietary recommendations emphasize the health and environmental benefits of transitioning to a more PB diet [53, 54]. Increasingly, U.S. consumers are replacing dairy with non-dairy alternatives, often due to perceived negative health effects [15, 16, 17]. Dairy is a top source of saturated fat [55], a nutrient Americans are encouraged to reduce in their diets for the prevention of cardiovascular disease [13, 56]. However, many studies have not found a direct association between dairy fat consumption and increased cardiovascular disease risk [21, 57, 58]. Studies have also found an inverse association between dairy fat consumption and risk of hyperglycemia [58] and type 2 diabetes [59, 60, 61]. Evidence suggests an association between dairy consumption and increased prostate cancer risk [62, 63, 64, 65], but also between dairy consumption and reduced risk of other types of cancers, like colorectal cancer [57, 66] and breast cancer [57, 67]. Despite promotion of dairy milk for bone health, research over the past two decades has not found significant evidence supporting calcium intake or milk intake for bone mineral density or reduced fracture risk [57, 68, 69, 70, 71] in adults, although intake does appear important during childhood and adolescence [57, 70]. Conclusions on the associations between dairy and certain health conditions have not yet been reached, yet U.S. consumers continue to reduce their dairy milk intake [8, 9]. However, fully omitting dairy foods can present nutritional issues [20], which many consumers may not be aware of [27, 28, 29, 30, 31].

Health professionals are trusted sources of nutrition information and the nutrition advice they give is likely to influence consumer food choices [4, 39]. This study found that many health professionals took a neutral stance on the overall health outcomes related to consuming both products, specifically stating that although dairy is a better source of nutrients, neither product is nutritionally superior to the other. Many health professionals also believed that consumers are confused about the nutritional differences between dairy products and their PB counterparts. However, dietetics professionals had a greater understanding of the nutrient adequacy of both products and believed more consumer confusion exists than other healthcare professionals. There appeared to be differences between the perceptions of health professionals who participated in the FDA’s request for comments and the survey sample of health professionals, suggesting beliefs among those participating in the federal rulemaking process may not reflect the beliefs of diverse health professionals in the U.S.

Aligning with prior research, we found that many U.S. health professionals recognized the beneficial nutrition qualities of dairy in one’s diet, in terms of essential nutrients, especially protein, calcium, and vitamin D [12, 14, 20], but also understood that PB alternatives have their own nutritional properties [10, 11, 12, 20] that may suit individual nutritional needs. About half of survey respondents reported that neither product was nutritionally superior. However, their open-ended responses reflected the belief that the dairy is a better source of essential nutrients. Although a greater proportion of Federal Register comments discussed the nutritional merits of PB dairy alternatives, about half of these only suggested that they are an *adequate* alternative for individuals who cannot or choose not to consume dairy, not necessarily stating that they are superior. For example, many Federal Register comments discussed that PB products may be beneficial for individuals with a dairy allergy or intolerance, or for those needing to reduce their saturated fat intake. Although their total nutritional makeup is not the same, and bioavailability differences may be present [14, 20] it is important to note that many PB beverages sold in the U.S. are fortified with calcium and vitamin D [14, 20, 72], and some PB products do have a considerable amount of protein [20, 72], providing consumers who do choose them with suitable alternatives. Survey respondents also cited dairy allergy or intolerance as a top reason that consumers may select a PB alternative. This aligns with literature showing that allergy, lactose intolerance and other digestive issues are a primary reason that consumers choose PB dairy alternatives [10, 12]. It may be that while health professionals see the positive aspects of PB alternatives, they would still recommend dairy as a first option if appropriate.

Over three-fourths of survey respondents were in agreement with dairy industry stakeholders [35, 36] that consumers do not understand the nutritional differences between dairy and PB alternatives, and two-thirds agreed that the current labeling, which allows PB products to use dairy terms like “milk”, “cheese”, and “yogurt” in their packaging, is contributing to this misunderstanding. A substantial minority of health professionals who submitted comments to the FDA and mentioned nutrition and health aspects also directly discussed consumer misunderstanding of the nutritional differences between the products, with some attributing this confusion to the current labeling regulations. Among all health professionals studied, we saw less strong opinions on the proposed labeling regulation change. Still, this adds to the limited evidence showing misconceptions about the nutritional properties of dairy and PB alternatives [27, 28, 29, 30, 31].

From the survey, significantly more non-dietetics professionals reported PB dairy alternatives to be nutritionally superior to dairy as compared to dietetics professionals. Dietetics professionals were more likely to believe that protein, vitamin D, and potassium may be lacking in the diets of individuals who do not consume dairy, and less likely to believe that no nutrients would be lacking. Many health professionals reported that diet is dependent on more than one food source, and that omitting dairy from the diet will not necessarily lead to nutrient inadequacies. However, 77% of health professionals also believed consumers do not understand the different nutritional aspects of both products, indicating that they may not know to consume other foods high in these nutrients. Likewise, data on the standard American diet [13, 73] shows that dairy is a top food source for nutrients of concern like calcium, vitamin D, vitamin A and potassium. Consumers who omit dairy but consume a balanced diet that includes fruits, vegetables, and seafood certainly may not be at risk for these inadequacies, however most Americans are not consuming enough of these food groups to be obtaining these nutrients through other sources [13]. Still, fewer non-dietetics professionals actually believed there is consumer confusion on the nutritional differences between products. These findings suggest that dietetics professionals may have a greater understanding of the role dairy has in the context of the American diet. As previous literature suggests [74, 75, 76, 77, 78, 79], nutrition training in many healthcare disciplines remains limited. RDNs receive extensive training in nutritional sciences and nutrition care, and may serve as important additions to interprofessional healthcare teams [77].

We also identified notable differences between health professionals from the survey sample and the Federal Register sample. A greater proportion (about one-third) of Federal Register participants discussed PB dairy alternatives being a nutritionally superior choice compared to less than 15% of survey respondents. Also, a much smaller proportion of health professionals who commented on the Federal Register believed consumers are confused about the nutritional differences, and/or that the use of the dairy terms in PB product labels is an issue. Likewise, almost two-thirds of health professionals who commented did not support a labeling regulation change, compared to less than 40% of health professionals from the survey. This may indicate that those who participated in this federal rulemaking process have more polarized opinions on the topic as compared to the general population of U.S. healthcare professionals.

This study had several imitations. First, there was an uneven distribution of health professional types within the survey sample, as the majority were dietetics and nursing professionals. However, having approximately half of respondents made up of dietetics professionals allowed us a large enough sub-sample to make a comparison of dietetics professionals to other health professionals with less nutrition training. Second, a large minority of the sample (32%) came from Vermont, a state with an agricultural economy centered on dairy production [51]. This allowed the novel and important inclusion of an independent variable to evaluate living in a state with a prominent dairy industry in our analyses. The majority of the sample was also female and non-Hispanic white, and nearly 85% were dietetics or nursing professionals. This reflects our additional recruitment activities targeted towards RDNs and nurses, and extended recruitment in Vermont. Over 90% of RDNs and nurses in the U.S. are female [80, 81] and less than 10% of the population of Vermont identify with a race or ethnicity other than non-Hispanic white [82]. The small sample size of our survey may have contributed to wide confidence intervals. Recoding during analysis to merge response options was performed to reduce this effect. The use of an online recruitment strategy, including on a social media platform, for our survey may have limited the range of healthcare professionals we were able to capture. However, less than half of our survey responses were generated from Facebook ad recruitment. Additionally, 85% of U.S. households now have internet access [83]. Household income is also associated with internet access [80], and the median annual wage for healthcare practitioners is about $70,000 [84], suggesting adequate reach to a range of health professionals. The use of qualitative data allowed us to capture more detailed and individualized responses from health professionals, although we were unable to confirm whether all comments came from health professionals from the U.S. Additionally, these data were coded using standard techniques for team-based research and it is possible that certain comments were interpreted incorrectly. However, all coders performed several rounds of preliminary practice to ensure agreement among coding and all codes were checked and discussed with at least one other person on the team.

## Conclusions

We found that many health professionals believe that consumers are confused about the nutritional differences between dairy and PB alternatives, and some believe that prohibiting the use of dairy terms in PB packaging may help reduce confusion. Our findings also indicate important knowledge gaps among non-dietetics health professionals around the nutrional adequacy of PB dairy alternatives, and the nutrional value dairy may have in the standard American diet. PB diets can be beneficial to health and longevity, but completely eliminating food groups can present nutritional challenges. Improved nutrition education and training focusing specifically on the nutritional needs of patients who follow PB dietary patterns may be necessary, to ensure health providers are equipped to help consumers make informed health decisions. Lastly, our findings suggest that stakeholders who submit comments to the Federal Register, which are then taken into account in the rulemaking process, may represent over-polarized views on nutrition issues.

## Supplementary Information

**Additional file 1.** 

**Additional file 2.** 

**Additional file 3.** 

## Data Availability

Data submitted to the U.S. Food and Drug Administration is publicly available at www.regulations.gov. Participant consent for the survey did not include consent for posting the data publicly. The data used are available from the corresponding author on reasonable request. The datasets used and/or analyzed during the current study are available from the corresponding author on reasonable request.

## Abbreviations

BIPOC: Black, Indigenous, or person of color
DO: Doctor of osteopathic medicine
FDA: Food and Drug Administration
LPN: Licensed practical nurse
MD: Medical doctor
NMPF: National Milk Producers Federation
NP: Nurse practitioner
PA: Physician assistant
PB: Plant-based
RDN: Registered dietitian nutritionist
RN: Registered nurse
