# Supplementary material for: Perspectives from healthcare professionals on the nutritional adequacy of plant-based dairy alternatives: results of a mixed methods inquiry
Source: BMC Nutr. 2022 May 12;8:46. doi: 10.1186/s40795-022-00542-7 (PMC9097167; doi:10.1186/s40795-022-00542-7)
Supplement: Supplementary file 1 — Additional file 1. [file 40795_2022_542_MOESM1_ESM.docx]

**
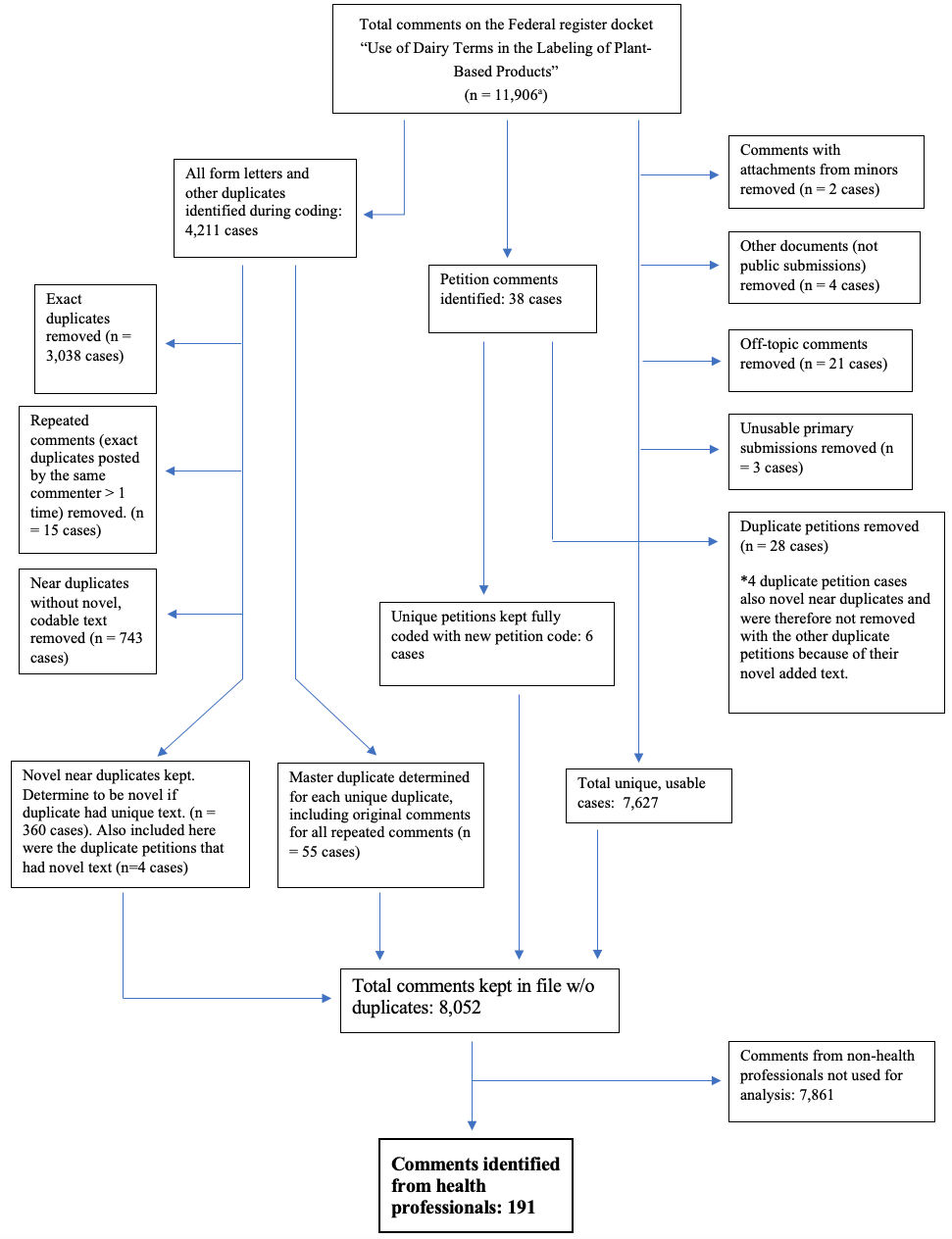
Additional File 1: Removal process for comment submissions not included in analysis.**

^a^ Originally 11,907; comment 11899 withdrawn by FR (late comment)
