# Supplementary material for: Perspectives from healthcare professionals on the nutritional adequacy of plant-based dairy alternatives: results of a mixed methods inquiry
Source: BMC Nutr. 2022 May 12;8:46. doi: 10.1186/s40795-022-00542-7 (PMC9097167; doi:10.1186/s40795-022-00542-7)
Supplement: Supplementary file 2 — Additional file 2. [file 40795_2022_542_MOESM2_ESM.docx]

**Additional File 2: Relevant codebook codes.**

| Code | Definition | When to use / When not to use | Examples | Source |
| --- | --- | --- | --- | --- |
| **COMMENT CHARACTERISTICS** |  |  |  |  |
| 4 – POSITION ON LABELING |  | Do not use.  One or more of the child codes MUST be applied to all comments. |  |  |
| 4.1 – SUPPORT MILK IN PB LABELING | Explicit support for labeling PB products with terms typically used to denote dairy products | Apply to full comment (select all). | “Totally fine, not confusing.”  “Yes, consumers are not stupid--we know what we're buying!”  “I do not believe that public is confused by the use of the word Milk in non dairy drinks.”  “I support the continued labeling of plant-based products with names that include milk, cultured milk, yogurt, and cheese.”  “I Want Milk From Plants, Not Abused Cows” | Original |
| 4.2 – OPPOSE MILK IN PB LABELING | Explicit opposition for labeling PB products with terms typically used to denote dairy products | Apply to full comment (select all). | “As a dairy farmer it is important to me that labeling and packaging of these alternative products is advertised truthfully. The ONLY way to accomplish this is to not use the term "milk" or "dairy" or any other terms referencing real dairy products when labeling these alternative products.”  “I believe that FDA needs to verify and enforce the these existing standards, and not permit plant-based beverages to be labeled as their dairy counterpart.”  “I am not a dairy farmer, but I am opposed to misleading labels.” | Original |
| 4.3 – UNCLEAR OR NEUTRAL POSITION ON PB LABELING | No explicit support or opposition for labeling PB products with terms typically used to denote dairy products | Apply to full comment (select all). |  | Original |
| 4.4 - SUPPORT OR OPPOSE DAIRY LABELING | Explicit support or opposition for labeling dairy products with special labels that would differentiate dairy products from PB products (e.g. “cow’s milk”). | Apply to full comment (select all). | “...if we're going to be petty why don't we demand that milk be labeled Cow Breast Milk.”  “...I do think that cows milk should carry a warning - like that on cigarettes - saying how much suffering has been caused by the manufacturing of the product and that it is a hazard to human health.”  “Labels on plant-based products aren't the problem the label on cow's milk is.”  “The liquid from a cow should properly be labeled "Mammary Discharge."”  “All the dairy industry has to do to avoid any confusion to consumers is to label milk-based products as such - for example "This product is made from cows' milk".” | Original |
| **COMMENT CONTENT** |  |  |  |  |
| 1 – NUTRITION AND HEALTH ASPECTS |  | Do not use. |  |  |
| 1.1 - CALCIUM | Calcium content of dairy or  products is noted as plant-based and/or the health implications of calcium consumption are noted | Apply to relevant text. | “If you look at what nutritional value of whole milk it is low in fat, high in protein and contains high levels of needed nutritional ingredients such as calcium…”  “While many consumers know that dairy products provide nine key nutrients necessary for healthy child development and adult health…” | Original |
| 1.2 - VITAMIN D | Vitamin D content of dairy or plant-based products is noted and/or the health implications of Vit D consumption are noted | Apply to relevant text. | “...vitamin A &D that plant based milk has to be added to come close to dairy milk.”  “While many consumers know that dairy products provide nine key nutrients necessary for healthy child development and adult health…” | Original |
| 1.3 - PROTEIN | The protein content of dairy or plant-based products is noted and/or the health implications of protein intake are noted | Apply to relevant text. | “In a recent survey, 73% of consumers believed that almond based drinks had as much or more protein per serving than milk,even though milk has 8 times as much protein.”  “If you look at what nutritional value of whole milk it is low in fat, high in protein and contains high levels of needed nutritional ingredients such as calcium…”  “While many consumers know that dairy products provide nine key nutrients necessary for healthy child development and adult health…” | Original |
| 1.4 - SUGAR | The sugar content of dairy or plant-based products is noted and/or the health implications of sugar intake are noted | Apply to relevant text. |  | Original |
| 1.5 - OTHER ESSENTIAL NUTRIENTS | Other macro or micro nutrients of dairy or plant-based products are mentioned and/or the health implications of other essential nutrients are noted  The general mention of PB or dairy products being rich in “nutrients”  This includes the mention of fiber  General mentions of the amount of fat and/or cholesterol, or the comparison of these amounts in PB or dairy products | Apply to relevant text. | “The vitamins, minerals, fat, and protein levels are different, which some consumers might not realize.”  “If you look at what nutritional value of whole milk it is low in fat, high in protein and contains high levels of needed nutritional ingredients such as calcium…”  “While many consumers know that dairy products provide nine key nutrients necessary for healthy child development and adult health…” | Original |
| 1.6 – OBESITY | Weight, BMI, or obesity | Apply to relevant text.  Use for references to weight implications or weight-loss implications of consuming dairy or plant-based products. |  | Original |
| 1.7 – DIGESTION | Breaking down or absorbing nutrients | Apply to relevant text.  Use for references to difficulty digesting dairy or plant-based products such as lactose intolerance, IBS, or generally not feeling well after consuming dairy or plant-based products. | “Dairy milk can cause a lot of gas in me, so I like having the option of using almond or rice milk, and I certainly don't confuse the two when I buy them or use them at home”  “People shouldn't be drinking cows milk. we do not need to drink dairy. We are unable to digest it after a certain age.” | Original |
| 1.8 – ALLERGY | Dairy or plant-based product allergies | Apply to relevant text.  Use for references to allergies to dairy or plant-based products.  Do NOT use for references to lactose intolerance. | “In fact, I am disappointed that some plant based "dairy" products (like "cheese") have casein in them. For me, that defeats my purpose”  “I think its just fine to call plant based milks - alternative milk. It makes it easier to find especially with those of us who have dairy allergies.” | Original |
| 1.9 – PRODUCT HEALTHFULNESS | General comments on how “healthy” or “unhealthy” PB products or dairy products are, about the superior health characteristics of either PB products or dairy products or when the nutritional differences between PB products and dairy are compared  Comments mentioning calories in PB or dairy milk or the comparison between the two | Apply to relevant text. | “The public is choosing delicious, healthy plant-based milk”  “Almond, Soy or other plant product Are healthier that's no confusion about it”  “I am glad that the plant based "dairy" products do not have the same nutritional profile as animal dairy products do, since, from my reading, regular consumption of animal milks are not healthy.”  “... I do think that cows milk should carry a warning - like that on cigarettes - saying how much suffering has been caused by the manufacturing of the product and that it is a hazard to human health”  “Dairy products are critical to the healthy diet of children and adults.”  “However, I feel that many may not realize or recognize the nutritional differences between dairy milk and these milk substitutes”  “This is clearly an effort by the dairy industry to protect their profits, as they face competition from healthier, more sustainable, and cruelty free plant-based dairy alternatives.” |  |
| 1.10 – HORMONES AND ANTIBIOTICS | Reference to use of growth hormones (e.g. rBST) or antibiotics |  |  |  |
| 1.11 – OTHER NUTR AND HEALTH | Reference to other nutrition or health conditions like diabetes, metabolic syndrome, infections, bone development (without mention of calcium or other nutrients), skin conditions, cancer, and heart disease  References to avoidance of saturated fat and/or cholesterol, unhealthfulness or negative health consequences of saturated fat and/or cholesterol, such as the mention of chronic disease related to their consumption | Apply to relevant text.  Do NOT use for general references to dairy or PB products being “healthy” or “healthier” than the other is.  See 1.9 – PB PRODUCT HEALTHFULNESS and 1.10 – DAIRY PRODUCT HEALTHFULNESS. |  | Original |
