# Supplementary material for: Perspectives from healthcare professionals on the nutritional adequacy of plant-based dairy alternatives: results of a mixed methods inquiry
Source: BMC Nutr. 2022 May 12;8:46. doi: 10.1186/s40795-022-00542-7 (PMC9097167; doi:10.1186/s40795-022-00542-7)
Supplement: Supplementary file 3 — Additional file 3. [file 40795_2022_542_MOESM3_ESM.docx]

**Additional File 3: Additional logistic regression analyses.**

Logistic regression analyses examined if health professional type is associated with the belief that the use of dairy terms in PB labeling is affecting consumer understanding, and the belief that the FDA should permit PB products to use dairy terms in their labeling (Supplementary Tables S1 and S2). No significant differences between dietetics professional and non-dietetics professional responses were observed.

Supplementary Table S1: Health professional type and belief on if PB product labeling is affecting consumer understanding (n=306).

|  | | Unadjusted analysis | | | | |  | Adjusted analysis | | | | |
| --- | --- | --- | --- | --- | --- | --- | --- | --- | --- | --- | --- | --- |
| PB labels affect consumer understanding | | b | SE | OR | CI | p value |  | b | SE | OR | CI | p value |
| Yes | |  |  |  |  |  |  |  |  |  |  |  |
|  | Dietetics professional (ref = non-dietetics professional) | -0.56 | 0.32 | 0.57 | 0.31-1.07 | 0.080 |  | -0.39 | 0.34 | 0.68 | 0.35-1.31 | 0.249 |
| No | |  |  |  |  |  |  |  |  |  |  |  |
|  | Dietetics professional (ref = non-dietetics professional) | -0.09 | 0.37 | 0.91 | 0.44-1.90 | 0.809 |  | -0.14 | 0.40 | 0.87 | 0.40-1.88 | 0.718 |

*Note*. Unadjusted and adjusted logistic regression models are presented above. The model used “unsure” as the reference category of the dependent variable. Adjusted models include age, race/ethnicity, and location (dairy state versus non-dairy state).

Supplementary Table S2: Health professional type and belief on if PB products should be permitted to use dairy terms (n=305).

|  | | Unadjusted analysis | | | | |  |  | Adjusted analysis | | | |
| --- | --- | --- | --- | --- | --- | --- | --- | --- | --- | --- | --- | --- |
| Allow dairy terms in PB labeling | | b | SE | OR | CI | p value |  | b | SE | OR | CI | p value |
| Yes | |  |  |  |  |  |  |  |  |  |  |  |
|  | Dietetics professional (ref = non-dietetics professional) | -0.28 | 0.28 | 0.76 | 0.44-1.31 | 0.323 |  | -0.21 | 0.30 | 0.81 | 0.45-1.45 | 0.476 |
| No | |  |  |  |  |  |  |  |  |  |  |  |
|  | Dietetics professional (ref = non-dietetics professional) | -0.31 | 0.28 | 0.97 | 0.56-1.69 | 0.912 |  | 0.28 | 0.31 | 1.32 | 0.72-2.43 | 0.365 |

*Note*. Unadjusted and adjusted logistic regression models are presented above. The model used “unsure” as the reference category of the dependent variable. Adjusted models include age, race/ethnicity, and location (dairy state versus non-dairy state).
